# Complete Genome Sequence of *Achromobacter* sp. Strain 77, a Hyphosphere Isolate of Fusarium oxysporum f. sp. *cucumerinum*

**DOI:** 10.1128/mra.01138-21

**Published:** 2022-03-31

**Authors:** Ji-Hong Li, Yu-Ling Jing, Rong-Jun Guo, Gui-Zhen Ma, Shi-Dong Li

**Affiliations:** a School of Environmental and Chemical Engineering, Jiangsu Ocean University, Jiangsu Lianyungang, China; b Institute of Plant Protection, Chinese Academy of Agricultural Sciences, Beijing, China; SIPBS, University of Strathclyde

## Abstract

The genome sequence of *Achromobacter* sp. strain 77, a bacterium isolated from the hyphosphere of Fusarium oxysporum f. sp. *cucumerinum*, is reported here. Genome sequencing and assembly yielded one chromosome consisting of 5,868,070 bases, with a G+C content of 65.89%.

## ANNOUNCEMENT

Hyphosphere bacteria of phytopathogenic fungi play important roles in plant growth and health (1). Here, soil samples from a maize field in China (116.71°E, 39.52°N) (5 to 20 cm in depth) were collected and used to grow cucumbers, and a rhizosphere soil bacterial suspension of cucumber was prepared using the filtration method (2). Hyphae of Fusarium oxysporum f. sp. *cucumerinum* were incubated in mineral medium at 20°C for 11 to 13 days, followed by the addition of a 2% soil bacterial suspension and incubation for another 24 h. By using an incubation slot method, 30 hyphosphere bacteria were isolated after 3 to 5 days of incubation on modified tryptic soy agar (TSA) at 28°C (2, 3). Among them, strain 77 was assigned to the genus *Achromobacter* based on the high level of identity (99.86%) of its 16S rRNA sequence, amplified by the universal primers 27F and 1492R (4), to that of Achromobacter mucicolens strain R-46658 (GenBank accession number NR_117613.1). Strain 77 intensively migrates along with the growth of F. oxysporum f. sp. *cucumerinum* hyphae and shows suppression ability against cucumber growth (5). Here, we report the genome sequence of strain 77 to reveal the genes related to its interactions with F. oxysporum f. sp. *cucumerinum* and plants.

For whole-genome sequencing of strain 77, 20 μg high-quality DNA (optical density at 260 nm [OD_260_]/OD_280_ of 1.9) was extracted from an overnight incubation in Luria-Bertani medium at 28°C using the Wizard genomic DNA purification kit (Promega) and sequenced at Shanghai Meiji. For Illumina sequencing, a library of 400- to 500-bp sheared fragments was prepared with the NEXTflex rapid DNA-sequencing (DNA-Seq) kit and paired-end sequenced (150-bp reads) on the HiSeq X Ten platform. For PacBio sequencing, genomic DNA was randomly sheared to ∼10-kb target size with g-TUBEs (Covaris), end repaired to prepare SMRTbell DNA template libraries according to the manufacturer’s instructions (Pacific Biosciences), and then purified using 0.45 volumes of Agencourt AMPure XP beads (Beckman Coulter Genomics, Danvers, MA). The generated ∼10-kb insert library was then sequenced on one single-molecule real-time (SMRT) Cell following standard methods. All sequence data were analyzed using the free online Majorbio Cloud platform v2 (https://cloud.majorbio.com). Default parameters were used for all software unless otherwise specified. After quality trimming (fastp v.0.20.1) (6), the Illumina and PacBio reads were assembled into a complete chromosome using Unicycler v0.4.7 (7) with default parameters. The genome was annotated using the NCBI Prokaryotic Genome Annotation Pipeline (PGAP) v4.12 (8).

A high-quality data set with a sequencing depth of 400× was generated. The *N*_50_ value of the PacBio raw reads was 15,807 bp, and 184,358 reads with an average length of 12,894 bp were generated. The Illumina sequencing platform generated 5,091,969 clean reads. The whole genome has 5,868,070 bases with a G+C content of 65.89% and is organized into a circular chromosome. Seventy-nine genes putatively coding for chemotaxis proteins and type VI secretion systems might contribute to the interactions with F. oxysporum f. sp. *cucumerinum* hyphae and plants (9–11).

### Data availability.

The whole-genome shotgun project was deposited at DDBJ/ENA/GenBank under the BioProject accession number PRJNA646886, the locus tag prefix H4P35 with the BioSample accession number SAMN15567587, and the accession number CP059848.1. The raw data have been deposited in the SRA database under the same BioProject accession number with the accession number SRP272884. The accession number of the 16S rRNA gene sequence of strain 77 is MN719982.1.
